# A Meta-Analysis of Health Status, Health Behaviors, and Health Care Utilization Outcomes of the Chronic Disease Self-Management Program

**DOI:** 10.5888/pcd10.120112

**Published:** 2013-01-17

**Authors:** Teresa J. Brady, Louise Murphy, Benita J. O’Colmain, Danielle Beauchesne, Brandy Daniels, Michael Greenberg, Marnie House, Doryn Chervin

**Affiliations:** Author Affiliations: Louise Murphy, Centers for Disease Control and Prevention, Atlanta, Georgia; Benita J. O’Colmain, Danielle Beauchesne, Brandy Daniels, Michael Greenberg, Marnie House, Doryn Chervin, ICF International, Atlanta, Georgia.

## Abstract

**Introduction:**

The Chronic Disease Self-Management Program (CDSMP) is a community-based self-management education program designed to help participants gain confidence (self-efficacy) and skills to better manage their chronic conditions; it has been implemented worldwide. The objective of this meta-analysis was to quantitatively synthesize the results of CDSMP studies conducted in English-speaking countries to determine the program’s effects on health behaviors, physical and psychological health status, and health care utilization at 4 to 6 months and 9 to 12 months after baseline.

**Methods:**

We searched 8 electronic databases to identify CDSMP-relevant literature published from January 1, 1999, through September 30, 2009; experts identified additional unpublished studies. We combined the results of all eligible studies to calculate pooled effect sizes. We included 23 studies. Eighteen studies presented data on small English-speaking groups; we conducted 1 meta-analysis on these studies and a separate analysis on results by other delivery modes.

**Results:**

Among health behaviors for small English-speaking groups, aerobic exercise, cognitive symptom management, and communication with physician improved significantly at 4- to 6-month follow-up; aerobic exercise and cognitive symptom management remained significantly improved at 9 to 12 months. Stretching/strengthening exercise improved significantly at 9 to 12 months. All measures of psychological health improved significantly at 4 to 6 months and 9 to 12 months. Energy, fatigue, and self-rated health showed small but significant improvements at 4 to 6 months but not at 9 to 12 months. The only significant change in health care utilization was a small improvement in the number of hospitalization days or nights at 4 to 6 months

**Conclusion:**

Small to moderate improvements in psychological health and selected health behaviors that remain after 12 months suggest that CDSMP delivered in small English-speaking groups produces health benefits for participants and would be a valuable part of comprehensive chronic disease management strategy.

## Introduction

The Chronic Disease Self-Management Program (CDSMP) is a 6-week community-based, self-management education program designed to help participants gain the confidence (self-efficacy) and skills to better manage their chronic conditions. It is taught by trained leaders who follow a structured protocol and given to participants who have various chronic conditions (1). Developed at Stanford University, the program has been disseminated throughout the United States and worldwide (1–4). A Spanish-language version (5) and alternative delivery modes, such as Internet-based “virtual” small-group classes (6), are also available.

The original CDSMP validation study found improvements in health status, health behaviors, and health care utilization (1). The findings of more recent studies, however, are inconsistent. For example, Lorig et al reported positive changes in self-rated health but not health distress (7), whereas Barlow et al found the opposite (8); Kennedy et al found positive changes in fatigue and exercise (2), but Goeppinger et al found neither (9). Summarizing the effect of CDSMP is difficult with such inconsistencies. Previous meta-analyses of chronic disease self-management programs suggest they offer modest health benefits (10–12), but these analyses combined multiple types of self-management programs, focused exclusively on randomized controlled trials (RCTs), and examined a limited range of outcomes.

The primary objective of this meta-analysis was to determine the short-term (4–6 months) and longer-term (9–12 months) effect of the Stanford CDSMP. We examined changes in health behaviors, physical and psychological health status (including self-efficacy), and health care utilization reported in CDSMP studies in English-speaking countries. A secondary objective was to determine whether program effect differed by delivery mode.

## Methods

### Data sources

We searched 8 electronic databases: Cochrane Central Register of Controlled Trials, Cochrane Database of Systematic Reviews, CINAHL, DARE, ERIC, EMBASE, Medline, and PsycINFO, to identify CDSMP studies published in peer-reviewed journals, online publications, and gray literature (ie, dissertations, conference abstracts, and unpublished reports) from January 1, 1999, through September 30, 2009. We used the following search terms: “arthritis,” “arthritis outcomes,” “arthritis patient education,” “arthritis program evaluation,” “arthritis self-help course,” “arthritis self-management,” “arthritis self-management program(me),” “ASMP,” “behavioral interventions,” “CDSMP,” “chronic disease self-management,” “chronic disease self-management program(me),” “osteoarthritis,” and “psychoeducational interventions.” We manually searched the reference lists of all studies located to identify other studies. Subject matter experts, identified as researchers who had studied chronic disease self-management interventions, and stakeholders, identified as representatives of organizations disseminating the Stanford CDSMP, provided feedback on the search and helped identify additional gray literature.

### Study selection

The study team developed and refined eligibility criteria using input from subject matter experts. Inclusion criteria were 1) intervention was the Stanford CDSMP, regardless of delivery mode; 2) CDSMP was implemented in an English-speaking country (United States, United Kingdom, Australia, Canada, or New Zealand), regardless of implementation language; 3) study examined at least 1 primary outcome from an RCT or pretest–posttest longitudinal program evaluation; and 4) report of findings was available in English. We excluded studies when 1) CDSMP was combined with another intervention, 2) intervention occurred in a non-English–speaking country, 3) program leaders did not use the requisite leaders’ manual, or 4) intervention included new content (beyond the requisite leaders’ manual).

Two reviewers (B.D., M.G.) conducted a 2-tiered eligibility review. A preliminary review focused on article abstracts; we excluded any study that did not meet all of the inclusion criteria or that met any exclusion criteria. If we could not determine eligibility from the abstract, we reviewed the full article. Of the original 146 studies identified, we were unable to obtain 12 despite multiple attempts to contact the author and publisher (Figure). The remaining 134 studies were screened; 34 met eligibility criteria (23% of the originally identified studies). We eventually eliminated an additional 11 studies because we were unable to retrieve data from the principal investigator (n = 3) or the study contained duplicative data (n = 8); the meta-analysis included 23 studies.

**Figure:**
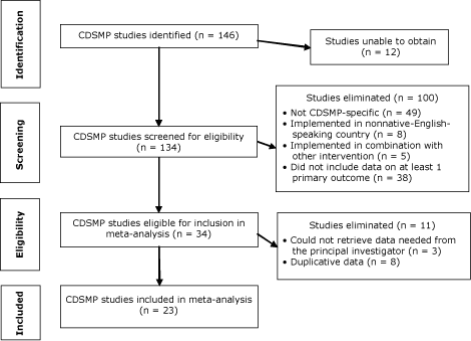
Selection of studies for meta-analysis of outcomes for the Chronic Disease Self-Management Program (CDSMP), 1999–2009.

### Data extraction

For each eligible article, the abstractors (B.D., M.G.) documented study design (RCT or longitudinal evaluation), participant characteristics, implementation factors (eg, delivery location [urban or rural, health care setting or community setting], leader characteristics [peer or professional, has or does not have chronic disease]), and study outcomes using a data abstraction tool and a codebook created to identify and code variables of interest. We categorized the studies according to delivery mode: small English-speaking group, small Spanish-speaking group, small group translated into other languages, Internet delivery, individual in-home delivery, or individual delivered over the telephone. To ensure abstraction consistency, the 2 reviewers both abstracted a randomly selected article each week; discrepancies were discussed until consensus was reached.

We inventoried the instruments used to measure each outcome in each study (Appendix A) and reviewed them for conceptual consistency. If an instrument was conceptually different from the other instruments measuring that outcome, we did not include the study in the meta-analysis for that outcome. We examined 20 outcomes in 4 domains: health behavior (aerobic exercise, cognitive symptom management [eg, prayer, meditation, muscle relaxation], communication with physician, and stretching/strengthening exercise); psychological health status (depression, health distress, and self-efficacy [overall, for chronic disease management, and for management of other symptoms]); physical health status (energy, fatigue, functional disability, pain, self-rated health, social role limitations, and shortness of breath), and health care utilization (emergency department visits, physician visits, hospitalization days or nights, and hospitalization times).

### Meta-analytic procedures

Because 18 of 23 studies reported on small English-speaking group interventions, we conducted 1 meta-analysis on this delivery mode and a separate meta-analysis on results by other delivery modes. In studies that evaluated 2 types of delivery, we analyzed each delivery mode as a separate study arm. For small English-speaking groups, we examined outcomes at 2 follow-up points: 4 to 6 months after baseline (short-term) and 9 to 12 months after baseline (longer-term). Among the small English-speaking group studies, 15 studies (5 RCT, 10 longitudinal) reported short-term outcomes; 7 studies (1 RCT, 6 longitudinal) reported longer-term outcomes. Because only 1 small English-speaking group RCT reported outcomes at 9 to 12 months, we examined pooled effect size (ES) by study design for 4- to 6-month outcomes only. The analysis by delivery mode at 4 to 6 months consisted of 15 small English-speaking groups and 7 other modes of delivery; this analysis was considered exploratory only because of the small number of studies on “other” delivery modes.

For each outcome, we generated an ES by combining the standardized difference in means from each eligible study. For longitudinal evaluations, the ES was the net difference between baseline and follow-up measures. For RCTs, the ES was the net difference between the intervention and control groups. We used a random-effects model, a more conservative approach that allows for both within-study and across-study variability, to derive ES. We standardized the sign (+,−) of the ES to the direction associated with a positive effect. For each outcome, the number of studies analyzed depended on the number of studies reporting that outcome. 

Whether data from studies can be appropriately pooled can be established by examining the degree of heterogeneity (ie, the extent to which outcomes vary across studies). We tested heterogeneity by using both the *Q* statistic and the *I* ^2^ statistic; a significant *Q* statistic (*P* ≤ .05) indicated significant heterogeneity (ie, a significant difference in ESs across studies). For the small English-speaking group studies, we tested variability in the overall ES for each outcome across studies and in the overall ES by study design (RCT vs longitudinal evaluation) because significant differences by study design would suggest that at least some of the intervention effect was attributable to the study design. In addition, we tested ES heterogeneity by delivery mode. We found no significant differences in ESs between RCTs and longitudinal studies for any of the 20 outcomes (Appendix B). Therefore, we calculated overall ESs for RCTs and longitudinal studies combined.

All analyses used Comprehensive Meta-Analysis version 2 (Biostat, Englewood, New Jersey) (13). Using the convention established by Cohen for social and behavioral science studies, we considered an ES of less than ±0.20 as small, ±0.20 to ±0.80 as moderate, and greater than ±0.80 as large (14). ES was statistically significant if *P* ≤ .05.

## Results

Twenty-five study arms from 23 studies were included in the meta-analysis; 2 studies reported on 2 delivery modes (Table 1). Most studies took place in the United States, Great Britain, or Canada. Whereas CDSMP was delivered in small English-speaking groups in 18 studies, 7 studies examined alternative delivery modes, including 2 small Spanish-speaking groups, a translation of the small English-speaking group into 4 languages, 2 Internet-delivery modes, and 2 individual in-home delivery modes (including 1 over the telephone). Twenty studies collected short-term follow-up data, and 11 studies collected longer-term follow-up data.

**Table 1:** Summary of Studies in Meta-Analysis of the Chronic Disease Self-Management Program (CDSMP), Studies Published 1999–2009

Study	Delivery Mode	Design for 4 –6-Month Analysis	Design for 9–12-Month Analysis	Follow-up Period
Barlow et al (8)	English-speaking small group	—	Longitudinal	1 y
Barlow et al (15)	English-speaking small group	RCT	RCT	4 mo; 1 y
Gitlin et al (16)	English-speaking small group	Longitudinal	—	4 mo
Goeppinger et al (9)	English-speaking small group	Longitudinal^a^	Longitudinal	4 mo; 1 y
Haas et al (17)	English-speaking small group	RCT	—	6 mo
Jerant et al (18)	1–1 in-person in home; 1–1 in-person over telephone	RCT	RCT	6 mo; 1 y
Kendall et al (19)	English-speaking small group	RCT	—	6 mo
Kennedy et al (2)	English-speaking small group	RCT	—	6 mo
Lorig et al (1)	English-speaking small group	RCT	—	6 mo
Lorig et al (20)	English-speaking small group	—	Longitudinal	1 y; 2 y
Lorig et al (21)	English-speaking small group	—	Longitudinal	1 y
Lorig et al (5)	Spanish-speaking small group	RCT	Longitudinal	4 mo; 1 y
Lorig et al (7)	English-speaking small group	Longitudinal^a^	Longitudinal	4 mo; 1 y
Lorig et al (22)	Spanish-speaking small group; English-speaking small group	Longitudinal	Longitudinal	4 mo; 1 y
Lorig et al (6)	Internet	RCT	RCT	6 mo; 1 y
Lorig et al (23)	Internet	Longitudinal	Longitudinal	6 mo; 1 y
McGowan (24)	English-speaking small group	Longitudinal	—	6 mo
McGowan (25)	English-speaking small group	Longitudinal	—	6 mo
McGowan (26)	English-speaking small group	Longitudinal	—	6 mo
McGowan (27)	English-speaking small group	Longitudinal	—	6 mo
McGowan (28)	English-speaking small group	Longitudinal	—	6 mo
Swerissen et al (29)	Small group translated into Chinese, Greek, Italian, and Vietnamese	RCT	—	6 mo
Wright et al (30)	English-speaking small group	Longitudinal	—	4 mo

Abbreviations: RCT, randomized controlled trial; —, no data reported.

a Study designed as an RCT to compare arthritis self-management program and CDSMP; data for CDSMP study arm at 4 to 6 months analyzed as longitudinal data (baseline to follow-up).

### Participants

Of the 8,688 participants included in all 23 studies, 2,909 were enrolled in RCTs and 5,779 in longitudinal studies. Three-quarters of participants were women. Among the 16 studies reporting participant age, participants were predominantly aged younger than 65 in 10 studies and 65 or older in 6 studies. For the 11 studies reporting education level, the mean number of years of education was 12.7. The interventions were conducted in primarily white populations; however, 2 studies focused on black populations, and 2 studies focused on Hispanic populations. The chronic disease diagnosis was confirmed by a physician in 6 study arms and self-reported in 19 study arms.

### ES at 4 to 6 months and 9 to 12 months

Of the 4 health behaviors studied among the small English-speaking groups, 3 showed significant small or moderate effects at 4 to 6 months: aerobic exercise (ES, 0.12); cognitive symptom management (ES, 0.26), and communication with physician (ES, 0.26) (Table 2). The improvements in aerobic exercise and cognitive symptom management remained significant at 9 to 12 months. Stretching/strengthening exercise showed no significant effects at 4 to 6 months but did show a small significant effect at 9 to 12 months.

**Table 2:** Overall Effect Size^a^ at 4 to 6 Months and 9 to 12 Months for English-Speaking Small Group Interventions (n = 22), Meta-Analysis of the Chronic Disease Self-Management Program, Studies Published 1999–2009

Outcome	4–6 Months (n = 15)		9–12 Months (n = 7)	
	No. of Studies (References)	Effect Size (95% CI)	No. of Studies (References)	Effect Size (95% CI)
**Health behavior**				
Aerobic exercise	9 (1,7,9,22,24–28)	0.12^b^ (0.04 to 0.19)	3 (7,9,21)	0.10^b^ (0.001 to 0.20)
Cognitive symptom management	9 (1,9,15,16,24–26,28,30)	0.26^b^ (0.16 to 0.36)	4(8,9,15,21)	0.37^b^ (0.26 to 0.48)
Communication with physician	8 (1,15,16,22,26–28,30)	0.26^b^ (0.08 to 0.42)	3 (8,15,21)	0.24 (−0.03 to 0.51)
Stretching/strengthening exercise	9 (1,7,9,16,24–28)	0.12 (−0.006 to 0.24)	2 (7,9)	0.15^b^ (0.007 to 0.30)
**Psychological health status**				
Depression^c^	6 (15,24–26,28,30)	−0.22^b^ (−0.30 to −0.13)	3 (8,15,21)	−0.21^b^ (−0.28 to −0.13)
Health distress^c^	12 (1,2,7,9,16,22,24–28,30)	−0.28^b^ (−0.36 to −0.20)	5 (7–9,20,21)	−0.23^b^ (−0.32 to −0.13)
Overall self-efficacy	5 (2,15,16,19,22)	0.35^b^ (0.19 to 0.49)	3 (15,20,21)	0.20^b^ (0.08 to 0.33)
Self-efficacy for chronic disease management	6 (7,24–26,28,30)	0.26^b^ (0.11 to 0.40)	2 (7,8)	0.38^b^ (0.04 to 0.71)
Self-efficacy for management of other symptoms	6 (24–28,30)	0.28^b^ (0.18 to 0.38)	1 (8)	0.45^b^ (0.29 to 0.61)
**Physical health status**				
Energy	8 (1,2,16,17,24–26,28)	0.16^b^ (0.02 to 0.29)	1 (20)	0.05 (−0.02 to 0.13)
Fatigue^c^	10 (7,9,15,22,24–28,30)	−0.14^b^ (−0.25 to −0.02)	5 (7–9,15,21)	−0.14 (−0.31 to 0.04)
Functional disability^c^	8 (1,7,9,16,24–26,28)	−0.06 (−0.01 to 0.05)	5 (7–9,20,21)	0.05 (−0.003 to 0.10)
Pain^c^	10 (7,9,15,22,24–28,30)	−0.13 (−0.27 to 0.01)	5 (7–9,15,21)	−0.13^b^ (−0.20 to −0.05)
Self-rated health	11 (1,2,7,9,16,22,24–28)	0.14^b^ (0.03 to 0.26)	4 (7,9,20,21)	−0.02 (−0.13 to 0.09)
Social role limitations^c^	9 (1,2,7,9,16,22,26–28)	−0.17^b^ (−0.21 to −0.12)	4 (7,9,20,21)	−0.14^b^ (−0.28 to −0.002)
Shortness of breath^c^	8 (1,22,24–28,30)	−0.08 (−0.84 to 0.40)	2 (8,21)	−0.10^b^ (−0.17 to −0.03)
**Health care utilization**				
Emergency department visits^c^	4 (16,22,26,27)	−0.02 (−0.08 to 0.05)	2 (20,21)	−0.10 (−0.22 to 0.02)
Physician visits^c^	9 (2,7,9,16,22,24,26,27,30)	−0.04 (−0.08 to 0.02)	4 (7,9,20,21)	−0.08 (−0.17 to 0.01)
Hospitalization days or nights^c^	8 (1,2,9,16,22,24,26,27)	−0.09^b^ (−0.15 to −0.02)	3 (9,20,21)	−0.04 (−0.09 to 0.02)
Hospitalization times^c^	7 (1,7,9,16,24,26,27)	−0.01 (−0.06 to 0.05)	4 (7,9,20,21)	−0.003 (−0.12 to 0.12)

Abbreviation: CI, confidence interval.

a Fifteen studies (5 randomized controlled trials and 10 longitudinal evaluations) were examined at 4 to 6 months and 7 studies (1 randomized controlled trial and 6 longitudinal evaluations) at 9 to 12 months. Effect size is the combination of the standardized differences in means for all eligible studies.

b *P* < .01, determined by *z* score test for significance of pooled effect size.

c Negative effect size indicates positive effect (eg, a decrease in depression).

We observed moderate significant effects for all psychological outcomes at 4 to 6 months and at 9 to 12 months; ESs ranged from 0.45 to −0.28. The strength of effects for depression and health distress were similar at both follow-up points. Whereas the effects for overall self-efficacy declined from the short-term to the long-term, they increased at 9 to 12 months for both self-efficacy for disease management and self-efficacy for management of other symptoms.

Changes in physical health outcomes were less consistent than changes in psychological outcomes. Energy, fatigue, and self-rated health showed small but significant improvements at 4 to 6 months but not at 9 to 12 months. Pain and shortness of breath did not change at 4 to 6 months but showed small significant improvements at 9 to 12 months. Functional disability did not change at either follow-up point. The small significant improvements in social role limitations at 4 to 6 months remained at 9 to 12 months.

The only significant change in health care utilization outcomes was a small improvement in the number of hospitalization days or nights at 4 to 6 months, but this improvement was not significant at 9 to 12 months.

### ES by delivery mode at 4 to 6 months

In the exploratory analysis by delivery mode at 4 to 6 months (Table 3), 6 of 12 outcomes examined in the small Spanish-speaking groups showed moderate significant improvements: aerobic exercise, health distress, overall self-efficacy, pain, self-rated health, and social role limitations.

**Table 3:** Overall Effect Size^a^ at 4 to 6 Months, by Delivery Mode, Meta-Analysis of the Chronic Disease Self-Management Program, Studies Published 1999–2009

Outcome	English-Speaking Small Group (n = 15)	Spanish-Speaking Small Group (n = 2)	Small Group Translation (n = 1)	Internet Delivery (n = 2)	Individual In-home (n = 2)	Between-Groups *P* Value^b^
**Health behavior**						
Aerobic exercise	0.12^c^	0.33^c^	—	0.09	—	.05
Cognitive symptom management	0.26^c^	—	0.39^c^	0.16	—	.54
Communication with physician	0.25^c^	0.16	—	0.22	—	.84
Stretching/strengthening exercise	0.12	—	—	0.17	—	.63
**Psychological health status**						
Depression^d^	−0.22^c^	—	−0.08	—	<.001	.06
Health distress^d^	−0.28^c^	−0.55^c^	−0.20	−0.26^c^	—	.17
Overall self-efficacy	0.34^c^	0.37^c^	0.39	0.24	0.14	.82
Self-efficacy for chronic disease management	0.26^c^	—	—	—	—	—
Self-efficacy for management of other symptoms	0.28^c^	—	—	—	—	—
**Physical health status**						
Energy	0.16^c^	—	0.39^c^	—	—	.23
Fatigue^d^	−0.15^c^	−0.12	−0.24^c^	−0.14^c^	—	.85
Functional disability^d^	−0.05	—	−0.08	−0.02	−0.001	.92
Pain^d^	−0.16^c^	−0.28^c^	—	−0.11^c^	—	.04
Self-rated health	0.14^c^	0.31^c^	0.39^c^	0.01	—	.16
Social role limitations^d^	−0.17^c^	−0.30^c^	−0.18	—	—	.06
Shortness of breath^d^	−0.13^c^	−0.20	−0.04	−0.12	—	.84
**Health care utilization**						
Emergency department visits^d^	−0.02	−0.10	−0.04	−0.06	—	.81
Physician visits^d^	−0.05	0.02	−0.12	−0.07	—	.87
Hospitalization days or nights^d^	−0.08^c^	−0.02	—	0.05	—	.17
Hospitalization times^d^	−0.01	—	—	−0.003	—	.92

Abbreviation: —, data not reported.

a Effect size is the combination of the standardized differences in means for all eligible studies.

b Determined by *Q* test for between-groups heterogeneity.

c *P* < .01, determined by *z *score test for significance of pooled effect size.

d Negative effect size indicates positive effect (eg, a decrease in depression).

Four of 12 outcomes examined in the small-group translation indicated moderate significant improvements: cognitive symptom management, energy, fatigue, and self-rated health. In the Internet-delivered intervention, 3 of 15 outcomes changed significantly; small improvements were evident for fatigue and pain, and moderate improvements were seen for health distress. Of the 3 outcomes examined in the individual in-home intervention, none changed significantly.

For all outcomes except pain, we found no significant differences in ES according to delivery mode. For pain, the small Spanish-speaking group showed a moderate improvement, whereas the small English-speaking group and the Internet delivery modes showed small improvements.

## Discussion

This study was a quantitative synthesis of 23 CDSMP studies to determine the effectiveness of CDSMP on health behaviors, physical and psychological health status, and health care utilization in both short-term and longer-term follow-up. The small English-speaking group delivery mode produced moderate improvements in self-efficacy and small and moderate improvements in psychological health and some health behaviors; many improvements were maintained for at least 12 months. Changes in physical health status were less consistent, and we found few significant changes in health care utilization. Our analysis is reasonably consistent with an analysis of lay-led self-management interventions (12); the differences between the analyses may have resulted from our analysis of CDSMP in isolation rather than in combination with other interventions and our inclusion of both RCTs and longitudinal evaluations. Our results are also consistent with small-group CDSMP studies conducted in Shanghai (31), Hong Kong (3,32), and Japan (33), although a Dutch study found no significant improvements from CDSMP (4). Our study also examined whether delivery mode influenced intervention effectiveness; our exploratory analysis suggested that alternative delivery modes are promising, although most alternative modes had fewer significant improvements than the small English-speaking group mode.

The benefits of CDSMP observed in this study have meaningful, wide-ranging, and complementary implications for chronic disease self-management and for primary and tertiary prevention of chronic disease. Analysis of the small English-speaking group delivery mode demonstrated consistent and sustained improvements in self-efficacy. Not only is self-efficacy the hypothesized mechanism of action for CDSMP (34) but it is also directly associated with such health behaviors as physical activity, healthful eating, pain-coping strategies, and medication adherence (35–37) and changes in pain, function, and depression (36). Although the ES was small, our study found direct changes in physical activity, an essential ingredient in primary and tertiary prevention of many chronic diseases (eg, arthritis, diabetes, heart disease) and crucial for general health and well-being (38). Interventions that increase physical activity among people who have chronic diseases are important tertiary prevention strategies. Reductions in health distress and depression are important benefits for people who have chronic diseases because depression is a common comorbidity (39) that complicates management of chronic diseases (40) and produces greater somatic symptoms and activity limitations (41,42). Interventions that improve these psychological outcomes can be useful adjuncts to clinical treatment.

The inconsistent changes in physical health status measures such as pain, fatigue, shortness of breath, and physical function may not be surprising. Because CDSMP is designed for people with various chronic health conditions, the presence and severity of symptoms like pain and shortness of breath varies among participants. Participants who rate symptoms as minimal at baseline have little room for improvement at follow-up. Changes in symptoms could be evaluated by segmenting participants’ symptom severity at baseline, but these data were not available in the reports we studied. However, a post-hoc subgroup analysis of a CDSMP RCT found that participants who reported lower self-efficacy, energy, and health-related quality of life at baseline reported greater benefits from CDSMP participation (43). Significant changes in pain and shortness of breath at 9 to 12 months that were not evident at 4 to 6 months may indicate a delay in improvement of some symptoms; however, energy and fatigue, which both improved in the short term, were no longer significantly improved in longer-term follow up. The improvements in social role limitations at both follow-up points may be a function of improvements in psychological distress; a study reported an association between psychological distress and limitations in people who have chronic diseases (42).

That we found only 1 small improvement in health care utilization may be due to several factors. First, CDSMP may not be sufficiently potent to produce decreases in an outcome as complex and multifactorial as health care utilization. Second, a healthy-participant bias may have affected our results: perhaps before attending CDSMP, participants had limited use of health care services, so little change was possible. Third, perhaps participants became more appropriate users of health care services (eg, those who were not seeking health care attention at advantageous times began to). Fourth, all measures of health care utilization used in these studies were based on self-report and may be insufficiently sensitive to identify changes. Using administrative claims data or contemporaneous reporting of utilization could provide more robust assessments of CDSMP effects on health care utilization.

This meta-analysis had several limitations. First, the unit of analysis was the study, not the individual. Factors such as comorbidity or symptom severity may confound estimates of intervention effectiveness at the study level. Second, for each outcome, the number of studies available for analysis varied, and some estimates were based on small sample sizes. We recommend caution when interpreting estimates based on small numbers of studies. The analysis by delivery mode at 4 to 6 months was exploratory because we examined only 7 studies that had delivery modes other than small English-speaking group. Finally, because our analysis focused on studies conducted in English-speaking countries and limited data were available on men and nonwhite racial/ethnic groups, our results may not be generalizable to other populations. 

This study also had several strengths. First, it is the only meta-analysis to examine the Stanford CDSMP alone and not in combination with other self-management or self-management education programs. Second, whereas most meta-analytic studies have examined only 2 to 4 outcomes per disease, we examined 20 outcomes. Third, the analyses examined data at 2 points postintervention to determine whether effects were maintained at longer-term follow-up. Finally, this is the first examination of the statistical validity of combining RCTs and longitudinal evaluations in analyses. We identified no heterogeneity by study design and determined that combining data from the 2 kinds of studies was appropriate; the combined analysis increases the generalizability of findings to populations most likely to enroll in CDSMP when it is offered in nonresearch settings.

This study identifies several areas for further research. Studies that differentiate among subpopulations would determine whether CDSMP is more effective in some populations than in others or whether contextual or implementation factors influence effectiveness. Additional studies are necessary to determine whether alternative delivery modes are as effective as the small English-speaking group mode. Direct measurement of health care utilization would provide more definitive data on CDSMP’s effect on health care utilization. A comparison of the effectiveness of CDSMP implemented alone and in combination with other self-management activities would also be useful. Finally, cost-effectiveness and cost-benefit analyses would help clarify the financial and quality-of-life return on investment of CDSMP.

The robust findings of small and moderate improvements in self-efficacy, psychological health, and select health behaviors that were maintained through 12 months suggest that the small English-speaking group CDSMP creates health benefits for program participants. The combined evidence from RCTs (with strong internal validity) and longitudinal program evaluations (with strong external validity) increases confidence that benefits will occur as programs are delivered in practice (44). Although some of the ESs obtained in this meta-analysis are modest, they have public health significance because of the cumulative effect of small changes in a large population — 141 million people in the United States have at least 1 chronic disease (45). CDSMP could have a considerable public health effect because of its potential scalability, low implementation cost, wide applicability across various settings and audiences, and capacity to reach large numbers of people.

CDSMP provides people who have chronic diseases with opportunities to develop the skills and confidence to self-manage their diseases and disease-related problems and improve their quality of life. On the basis of our meta-analysis, health care systems and community organizations can adopt CDSMP as part of their comprehensive chronic disease management strategy to increase their constituents’ psychological health status, physical activity, and confidence in their ability to manage their chronic conditions, and health care providers can confidently recommend CDSMP to achieve these same benefits in their patients with chronic disease. The self-management supports that communities and health systems provide, such as CDSMP, are essential components of patient-centered medical homes (1) and the chronic-care model (46) that is reshaping how care is delivered to people who have chronic health conditions.

## Appendix A. Inventory of Instruments Used to Measure Outcomes in Meta-Analysis of Studies on the Chronic Disease Self-Management Program, Studies Published 1999–2009

**Table:**

Outcome	Instrument
**Health behavior**	
Aerobic exercise	Self-report of number of times per week doing aerobic exercise
Cognitive symptom management	Stanford Patient Education Research Center scale (http://patienteducation.stanford.edu/research/cognitivesymptom.html) adapted from the Medical Outcomes Study (MOS) (http://www.rand.org/health/surveys_tools/mos/mos_core_36item.html)
Communication with physician	Stanford Patient Education Research Center scale (http://patienteducation.stanford.edu/research/communprov.html)
Stretching/strengthening exercise	Self-report of number of times per week doing stretching/strengthening exercise
**Psychological health status**	
Depression	Depression subscale of the Hospital Anxiety and Depression Scale (http://www.ncbi.nlm.nih.gov/pubmed/6880820) or Center for Epidemiological Studies Depression Scale (http://cesd-r.com/)
Health distress	Stanford Patient Education Research Center scale adapted from the MOS (http://patienteducation.stanford.edu/research/healthdistress.html)
Overall self-efficacy	Stanford Patient Education Research Center’s Chronic Disease Self-Efficacy Scales (31-item or 6-item version). Some principal investigators selected groups of items from the 32-item version (http://patienteducation.stanford.edu/research/secd32.html; http://patienteducation.stanford.edu/research/secd6.html)
Self-efficacy for chronic disease management	5-item Manage Disease in General subscale of the Stanford Patient Education Research Center’s Self-Efficacy for Managing Chronic Disease (http://patienteducation.stanford.edu/research/secd32.html)
Self-efficacy for management of other symptoms	5-item Manage Symptoms subscale of the Stanford Patient Education Research Center’s Self-Efficacy for Managing Chronic Disease (http://patienteducation.stanford.edu/research/secd32.html)
**Physical health status**	
Energy	Stanford Patient Education Research Center 5-item energy/fatigue scale from the MOS (http://patienteducation.stanford.edu/research/energyfatigue.html)
Fatigue	10-point visual analog or visual numeric scale
Functional disability	Health Assessment Questionnaire–Disability scale (http://aramis.stanford.edu/HAQ.html); Western Ontario and McMaster University Osteoarthritis Index (http://www.womac.org/)
Pain	10-point visual analog or visual numeric scale; McGill present pain intensity rating (http://tuum-est.com/pdf/rsd/the-mcgill-pain-questionnaire-from-description-to-measurement-melzack.pdf)
Self-rated health	1 question from the National Health Interview Survey or the MOS (http://www.rand.org/health/surveys_tools/mos/mos_core_36item.html); scale scoring was reversed in our meta-analysis for ease of interpretation
Shortness of breath	10-point visual analog or visual numeric scale
Social role limitations	Stanford Patient Education Research Center scale adapted from the MOS (http://patienteducation.stanford.edu/research/activitieslimit.html)
**Health care utilization**	
Emergency department visits	Self-report of the number of emergency department visits in past 4 to 6 months
Physician visits	Self-report of the number of physician visits in past 4 to 6 months
Hospitalization days or nights	Self-report of the number of days or nights hospitalized in the past 6 months
Hospitalization times	Self-report of the number of times hospitalized in the past 6 months

## Appendix B. Test of Heterogeneity of Effect Size by Study Design^a^ at 4 to 6 Months for English-Speaking Small-Group Intervention, Meta-Analysis of the Chronic Disease Self-Management Program, Studies Published 1999–2009

**Table:**

Outcome	Randomized Controlled Trial		Longitudinal Evaluation		Between-Groups* P *Value^c^
	n^b^	Effect Size (95% CI)	n^b^	Effect Size (95% CI)	
**Health behavior**					
Aerobic exercise	1	0.20 (0.005 to 0.40)^d^	8	0.11 (0 to 0.18)^d^	.38
Cognitive symptom management	2	0.31 (0.09 to 0.53)^d^	7	0.25 (0.14 to 0.35)^d^	.61
Communication with physician	2	0.15 (−0.20 to 0.51)	6	0.29 (0.09 to 0.48)^d^	.51
Stretching/strengthening exercise	1	0.14 (−0.20 to 0.49)	8	0.11 (−0.01 to 0.24)	.86
**Psychological health status**					
Depression^e^	1	−0.18 (−0.52 to 0.18)	5	−0.22 (−0.30 to −0.13)^d^	.81
Health distress^e^	2	−0.22 (−0.42 to −0.01)^d^	10	−0.30 (−0.39 to −0.20)^d^	.47
Overall self-efficacy	3	0.43 (0.21 to 0.64)^d^	2	0.28 (0.08 to 0.47)^d^	.29
Self-efficacy for chronic disease management	0	NA	6	0.26 (0.11 to 0.40)^d^	NA
Self-efficacy for management of other symptoms	0	NA	6	0.28 (0.18 to 0.38)^d^	NA
**Physical health status**					
Energy	3	0.21 (−0.02 to 0.44)	5	0.13 (−0.03 to 0.29)	.58
Fatigue^e^	1	0.03 (−0.30 to 0.37)	9	−0.16 (−0.21 to −0.10)^d^	.27
Functional disability^e^	1	−0.15 (−0.33 to 0.03)	7	−0.03 (−0.10 to 0.05)	.22
Pain^e^	1	0.04 (−0.29 to 0.37)	9	−0.16 (−0.21 to −0.12)^d^	.23
Self-rated health	2	0.15 (−0.10 to 0.41)	9	0.14 (0.01 to 0.27)^d^	.93
Social role limitations^e^	2	−0.21 (−0.31 to −0.10)^d^	7	−0.16 (−0.20 to −0.11)^d^	.41
Shortness of breath^e^	1	0.05 (−0.18 to 0.28)	7	−0.16 (−0.25 to −0.06)^d^	.10
**Health care utilization**					
Emergency department visits^e^	0	NA	4	−0.02 (−0.08 to 0.05)	NA
Physician visits^e^	1	−0.01 (−0.19 to 0.17)	8	−0.04 (−0.09 to 0.02)	.78
Hospitalization days or nights^e^	2	−0.14 (−0.24 to −0.04)^d^	6	−0.06 (−0.12 to −0.002)^d^	.21
Hospitalization times^e^	1	−0.02 (−0.16 to 0.12)	6	−0.01 (−0.07 to 0.06)	.82

Abbreviations: CI, confidence interval; NA not applicable.

^a ^Effect size is the combination of the standardized differences in means for all eligible studies. Whether data from studies can be appropriately pooled can be established by examining the degree of heterogeneity (ie, the extent to which outcomes vary across studies). We tested heterogeneity by using both the *Q* statistic and the *I* ^2^ statistic; a significant *Q* statistic (*P* ≤ .05) indicated significant heterogeneity (ie, a significant difference in effect sizes across studies).

^b^ Number of studies included in analysis.

^c^ Determined by *Q* test for between groups heterogeneity.

^d^ *P* < .01, determined by *z* score test for significance of pooled effect size.

^e^ Negative effect size indicates positive effect (eg, a decrease in depression).
